# Application of Remote Follow-Up Via the WeChat Platform for Patients who Underwent Congenital Cardiac Surgery During the COVID-19 Epidemic

**DOI:** 10.21470/1678-9741-2020-0256

**Published:** 2021

**Authors:** Qi-Liang Zhang, Shu-Ting Huang, Ning Xu, Zeng-Chun Wang, Hua Cao, Qiang Chen

**Affiliations:** 1 Department of Cardiac Surgery, Fujian Maternity and Child Health Hospital, Affiliated Hospital of Fujian Medical University, Fuzhou, China.; 2 Fujian Key Laboratory of Women and Children’s Critical Diseases Research, Fujian Maternity and Child Health Hospital, Fuzhou, China.; 3 Department of Cardiovascular Surgery, Union Hospital, Fujian Medical University, Fuzhou, China.

**Keywords:** COVID-19, Social Media, Attitude to Health, Cardiovascular System, China, Severe Acute Respiratory coronavirus 2

## Abstract

**Objective:**

To investigate the effect of WeChat-based telehealth services on the postoperative follow-up of children who underwent congenital heart surgery during the COVID-19 epidemic.

**Methods:**

This study retrospectively analyzed the clinical and family data of 108 children who underwent congenital heart surgery and underwent remote follow-up via the WeChat platform from December 2019 to March 2020 in our hospital.

**Results:**

During the follow-up period, the WeChat platform was used to refer 8 children with respiratory infection symptoms to local hospitals for treatment. Two children with poor incision healing were healed after we used the WeChat platform to guide the parents in dressing the wounds on a regular basis at home. Nutritional guidance was given via the WeChat platform to 13 patients with poor growth and development. The psychological evaluation results of the parents showed that the median (range) SDS score was 43 (34-59), and 7 parents (6.5%) were classified as depressed; the median (range) SAS score was 41 (32-58), and 12 parents (11.1%) were classified as having mild anxiety.

**Conclusion:**

The use of WeChat-based telehealth services was effective for the remote postoperative follow-up of children who underwent congenital cardiac surgery during the COVID-19 epidemic. Providing WeChat-based telehealth services can reduce the amount of travel required for these children and their families, which is helpful for controlling and preventing the spread of COVID-19.

**Table:** 

Abbreviations, acronyms & symbols	
**CHD**	**= Congenital heart disease**
**COVID-19**	**= Coronavirus disease 2019**
**SARS**	**= Severe acute respiratory syndrome**
**SAS**	**= Self-Rating Anxiety Scale**
**SDS**	**= Self-Rating Depression Scale**

## INTRODUCTION

Recently, a new coronavirus disease (COVID-19) emerged and spread quickly in China and around the world^[^^1^^]^. Although strong measures have been taken to control the virus, there were still a large number of people infected, and some of them died because of ineffective treatment^[^^2^^-^^6^^]^. During this particular period, people with congenital heart disease (CHD) still had to undergo surgical correction and needed close medical monitoring. The poor physical quality and immune functions of children who underwent congenital cardiac surgery made them more susceptible to respiratory infection than normal people. However, regular monitoring of these patients was required to assess their postoperative recovery. Due to the uneven distribution of medical resources, many patients living in remote areas have to travel to hospitals in central cities in China to undergo cardiac surgery. There was a high risk of respiratory infection in the hospital and while traveling to the hospital, as this travel might increase population mobility and the spread of the disease. For patients, if they came to the hospital for a follow-up visit as scheduled, they would face the risk of being infected by the virus; if they did not come to the hospital, they would worry that any subsequent heart problems might not be addressed in time. These options led to a heavy mental burden for the parents. Therefore, providing effective telehealth services for the postoperative follow-up of children who underwent congenital cardiac surgery has become extremely important during the COVID-19 epidemic. The purpose of this study was to explore the effects of using the WeChat platform on the remote postoperative follow-up of these patients during the epidemic.

## METHODS

The present study was approved by the ethics committee of Fujian Medical University, China, and adhered to the tenets of the Declaration of Helsinki. Additionally, all parents signed the consent form before participating in the study.

### Patients

This study retrospectively analyzed the clinical and family data of 108 children who underwent congenital cardiac surgery and remote follow-up via the WeChat platform from December 2019 to March 2020 at our hospital. All patients’ underlying diseases were assessed: 32 cases of atrial septal defect, 46 cases of ventricular septal defect, 13 cases of patent ductus arteriosus, 12 cases of pulmonary stenosis, and 5 cases of tetralogy of Fallot. Other relevant clinical data are shown in Table 1.

**Table 1:** Patient demographics or patient characteristics.

Item	
Number	108
Age, median (range)	2.8 years (1 month-9 years)
Weight, median (range)	12.4 (3.2-37.6) kg
Disease	
Atrial septal defect	32
Ventricular septal defect	46
Patent ductus arteriosus	13
Pulmonary stenosis	12
Tetralogy of Fallot	5
Parents' age, median (range)	29 years (22-43) years
Parents' education level	
Under high school	20
High school	42
Junior college	31
Bachelor degree or higher	15
Living condition	
Rural area	76
City	32

### WeChat-Based Telehealth Services

The content of the telehealth service provided via the WeChat platform mainly included two parts: the education module and the answering and solving questions module. The education module included relevant knowledge about CHD, postoperative care and nutrition, and the management of related postoperative complications. Parents could review and learn these materials at their convenience. Questionnaires were distributed through the WeChat platform to assess the psychological status of the parents of children who underwent congenital cardiac surgery during the COVID-19 epidemic. This questionnaire mainly included the Self-Rating Depression Scale (SDS) and the Self-Rating Anxiety Scale (SAS). In addition to the structured questionnaire, parents were allowed to express any other concerns. In the answering and solving questions module, one medical staff member was available from 8 am to 21 pm every day to answer parents’ questions, send reminders and supervise regular outpatient reviews, and remind parents about the possibility of postoperative complications due to delayed treatment. The medical staff also encouraged the family members to actively discuss the care experience with each other in the WeChat group.

### Research tools

1. SDS was applied in this study^[^^7^^]^. This scale consists of 20 items, including 10 negative symptoms and 10 positive symptoms. The items assess the respondent’s mood, body discomfort symptoms, spiritual movement, behavior, and psychological symptoms. The response options for each item range from 1 to 4, with higher scores indicating a higher frequency of symptoms (the items were reverse scored to assess the frequency of negative symptoms). The standardized score was obtained by multiplying the scores by 1.25 and rounding the result. Normally, the maximum score was 41 and the maximum standardized total score was 53. Higher scores indicate a trend towards more significant depression. Grade description: <50 indicates normal, 50 to 59 indicates mild depression, 60 to 69 indicates moderate depression, and ≥70 indicates severe depression.

2. SAS was applied in this study^[^^8^^]^. This scale mainly evaluated the subjective patients’ feeling of anxiety, being a self-reported tool. SAS has been extensively applied in clinics and is characterized by high reliability and validity. The scale included 15 items with negative words and five items with positive words. The response options for each item range from 1 to 4, with higher scores indicating a higher frequency of symptoms (the items were reverse scored to assess the frequency of the positive symptoms). The total score was obtained by summing the scores of all items. The standardized score was obtained by multiplying the total score by 1.25 and rounding the result. The mean value of the standardized score is 50. Grade description: <50 indicates normal, 50 to 59 indicates mild anxiety, 60 to 69 indicates moderate anxiety, and ≥70 indicates severe anxiety.

## RESULTS

A total of 108 children who underwent congenital cardiac surgery participated in this study. The median age of the children was 2.8 years, and ages ranged from 1 month to 9 years. The median weight was 12.4 kg, and the weights ranged from 3.2 kg to 37.6 kg. A total of 70.4% (76/108) of the children lived in rural areas, and 57.4% (62/108) of the parents had high school education or lower education level (Table 1).

Eight children with respiratory infection symptoms were referred to local hospitals via the WeChat platform. These patients were diagnosed with community-acquired pneumonia at the local hospital through symptoms, signs and tests and were also excluded from specific bacterial or viral infections. They were easily cured with conventional drugs. Two children with poor incision healing were healed after we used WeChat to guide the parents in dressing the wounds on a regular basis at home. Nutritional guidance was given via the WeChat platform to 13 patients with poor growth and development. No child suffered from arrhythmia or heart failure (Table 2). The parents’ psychological evaluations showed that the median (range) SDS score was 43 (34-59), and 7 parents (6.5%) were classified as depressed; the median (range) SAS score was 41 (32-58), and 12 parents (11.1%) were classified as having mild anxiety (Table 3).

**Table 2:** Postoperative complications of children.

Item	
Respiratory infections	8
Poor growth and development	13
Poor incision healing	2
Arrhythmia	0
Heart failure	0

**Table 3:** Parents' psychological status.

Item	
SDS score, median (range)	43 (34-59)
Normal (score: <50)	101
Mild depression (score: 50-59)	7
Moderate depression (score: 60-69)	0
Severe depression (score: >70)	0
SAS score, median (range)	41 (32-58)
Normal (score: <50)	96
Mild anxiety (score: 50-59)	12
Moderate anxiety (score: 60-69)	0
Severe anxiety (score: >70)	0

## DISCUSSION

CHD is the most common congenital structural deformity; 2-3% of newborns suffer from CHD every year, and this deformity may be life-threatening if not treated^[^^9^^-^^11^^]^. Surgical correction is the main method to treat CHD. Children with congenital cardiac surgery are usually affected by cardiac malformations, and their physical and immune functions are often worse than those of normal people. Therefore, children who undergo congenital cardiac surgery often need close follow-up and regular monitoring to assess their physical condition and postoperative cardiac recovery after discharge.

The COVID-19 epidemic broke out in China and spread rapidly across the country and the world in December 2019^[^^12^^]^. To effectively prevent and control the epidemic, the Chinese government has taken strict measures such as blocking cities and roads to limit the flow of people, encouraging all residents to stay at home as much as possible and restricting social contact to cut transmission routes and reduce the spread of the virus^[^^13^^]^. During this epidemic period, only a limited portion of the urban public transport system was working, and all interprovincial public transport routes were discontinued. However, advanced medical care is mainly concentrated in some large cities in China, and the primary health facilities are relatively scarce; thus, the number of doctors able to perform cardiac surgery is low. However, most patients with CHD live in rural areas, and the patients’ parents mostly have secondary education or lower education level, so they have little knowledge about CHD and are unable to fully understand the information about the disease during hospitalization. In this study, 70.4% of the children lived in rural areas far from the city, and 57.4% of the patients’ parents had secondary education or lower education level. Many parents might feel pressure and anxiety about postoperative family care and find it difficult to deal with unexpected events. During this epidemic, it was very inconvenient for people to travel for medical treatment, especially people living in rural areas, and this travelling might increase the risk of virus infection. A lack of medical knowledge and difficulty in seeking medical support would greatly increase the pressure and anxiety among the parents of children who underwent congenital cardiac surgery. Therefore, it was particularly important to implement a new method to remote follow-up with these patients and to improve telehealth services, which could provide patients with medical service support and facilitate the prevention and control of COVID-19.

Smartphones have been widely used in recent years, especially in China^[^^14^^]^. Many different social media platforms have been widely used in health management and in disease-related education^[^^15^^,^^16^^]^. Unlike other social media platforms, the WeChat platform can be used for graphs, text, audio, video and so on. It is a popular, convenient, interactive and intuitive way of exchanging information, and thus, it is an important platform for maximizing information coverage and effectiveness^[^^17^^]^. In addition, health education via the WeChat platform has been found to be very effective in saving time, reducing economic costs, improving treatment adherence, reducing complications, increasing follow-up rates and improving patients' conditions^[^^18^^-^^20^^]^. During the COVID-19 epidemic, we provided remote follow-up and telehealth services through the WeChat platform for children who underwent congenital cardiac surgery. The parents of these children could acquire knowledge from the WeChat education module anytime and anywhere according to their needs. When there was a problem, they could consult the medical staff through WeChat so that they could obtain timely and effective answers from professionals. In this study, 8 children with respiratory infections were referred to local hospitals after receiving timely advice via the WeChat platform. Two children with poor incision healing were cured after we used WeChat video to guide the parents in dressing the wounds at home. We provided nutritional guidance through the WeChat platform to 13 patients with poor growth and development. In this way, the parents could obtain medical support without leaving home, which reduced the need to travel for medical care during the COVID-19 epidemic.

Infectious diseases not only affect the physical health of patients but also affect the mental health and well-being of uninfected people^[^^21^^]^. Previous studies have shown that an increase in the prevalence of infectious diseases, such as severe acute respiratory syndrome (SARS), leads to increases in the levels of anxiety, depression and stress in the population^[^^22^^-^^23^^]^. Studies have also shown that people with health problems and their families who needed to go to the clinic were vulnerable to anxiety and depression, and the panic and anxiety caused by the pandemic of COVID-19 would further exaggerate these conditions^[^^24^^]^. During this epidemic, due to the inconvenience of traveling for hospital visits, patients could not return to the hospital for timely follow-up visits. The parents were worried about the cardiac recovery of the child, especially when the child had symptoms such as respiratory tract infection, poor growth and development, and poor wound healing. Through the WeChat platform, parents could directly contact the medical staff and easily obtain support from doctors, thus alleviating their concerns in a timely manner and reducing their anxiety and depression. Additionally, this method allowed us to provide psychological counseling and support to parents with high levels of stress or anxiety. In this study, most parents did not experience anxiety and depression, and those who experienced anxiety and depression showed only mild symptoms.

This article has some limitations. First, this study was a single-center retrospective study with a small number of cases. Second, we did not have a control group, so this was a descriptive study. Third, this was a study performed during an epidemic, and the results might not apply to other periods or might require further elaboration. Fourth, we did not discuss the relationship between parents’ psychological status and the postoperative outcomes, patient’s age, or patient’s injuries in this study.

## CONCLUSION

During the COVID-19 epidemic, remote follow-up and telehealth services through the WeChat platform for children who underwent congenital cardiac surgery can provide a timely assessment of the physical status of children, and this platform allows practitioners respond to problems in the process of family care and provide guidance for home care, which relieves stress, anxiety and depression of the parents of these patients. This method can also reduce the need for travel for these children and their families, which is helpful for controlling and preventing the spread of COVID-19.

**Table:** 

Authors' roles & responsibilities	
Q-LZ	Substantial contributions to the conception or design of the work; or the acquisition, analysis or interpretation of data for the work; drafting the work or revising it critically for important intellectual content; final approval of the version to be published
S-TH	Substantial contributions to the conception or design of the work; or the acquisition, analysis or interpretation of data for the work; final approval of the version to be published
NX	Substantial contributions to the conception or design of the work; or the acquisition, analysis or interpretation of data for the work; final approval of the version to be published
Z-CW	Substantial contributions to the conception or design of the work; or the acquisition, analysis or interpretation of data for the work; final approval of the version to be published
HC	Substantial contributions to the conception or design of the work; or the acquisition, analysis or interpretation of data for the work; drafting the work or revising it critically for important intellectual content; final approval of the version to be published
QC	Substantial contributions to the conception or design of the work; or the acquisition, analysis or interpretation of data for the work; final approval of the version to be published
